# Hearing difficulty is linked to Alzheimer’s disease by common genetic vulnerability, not shared genetic architecture

**DOI:** 10.1038/s41514-021-00069-4

**Published:** 2021-07-22

**Authors:** Fatin N. Zainul Abidin, Helena R. R. Wells, Andre Altmann, Sally J. Dawson

**Affiliations:** 1 UCL Ear Institute, University College London, London, UK; 2 Centre for Medical Image Computing, Department of Medical Physics and Biomedical Engineering, University College London, London, UK; 3 Department of Twin Research and Genetic Epidemiology, King’s College London, London, UK

**Keywords:** Neurological disorders, Alzheimer's disease, Auditory system

## Abstract

Age-related hearing loss was recently established as the largest modifiable risk factor for Alzheimer’s disease (AD), however, the reasons for this link remain unclear. We investigate shared underlying genetic associations using results from recent large genome-wide association studies (GWAS) on adult hearing difficulty and AD. Genetic correlation and Mendelian randomization (MR) analysis do not support a genetic correlation between the disorders, but suggest a direct causal link from AD genetic risk to hearing difficulty, driven by *APOE*. Systematic MR analyses on the effect of other traits revealed shared effects of glutamine, gamma-glutamylglutamine, and citrate levels on reduced risk of both hearing difficulty and AD. In addition, pathway analysis on GWAS risk variants suggests shared function in neuronal signalling pathways as well as etiology of diabetes and cardiovascular disease. However, after multiple testing corrections, neither analysis led to statistically significant associations. Altogether, our genetic-driven analysis suggests hearing difficulty and AD are linked by a shared vulnerability in molecular pathways rather than by a shared genetic architecture.

## Introduction

Dementia and age-related hearing loss (*ARHL*) are two of the most common age-related diseases, affecting ~50 million and 466 million worldwide, respectively^1,2^. Both have a significant impact on the health and wellbeing of the ageing population^3–5^ causing social isolation, higher healthcare costs, and currently have limited treatment options. Alzheimer’s disease (AD) is the most common type of dementia causing progressive memory loss, including at later stages severe cognitive dysfunction affecting basic language and thinking skills. The biological hallmarks of *AD* are the accumulation of extracellular amyloid β-peptide (Aβ) plaques and intracellular neurofibrillary tangles, extensive neuronal loss, brain atrophy, and inflammation. Aβ plaques appear to be central to initiating AD, whereas neurofibrillary tangles correlate with the progression of *AD*^6^. *ARHL* or presbycusis is the most common form of sensory loss in older people and the third most common health condition in older adults after heart disease and arthritis^7^. It is characterized by bilateral hearing thresholds of >25 dB, often affecting the higher frequencies first before progressing to lower frequencies, as well as hearing difficulty with background noise^2,8^. The underlying pathological mechanisms responsible for ARHL are not well understood but are likely to involve a combination of pathologies acquired in different components of the highly complex auditory pathway.

Recently, various studies have linked the incidence of dementia and hearing loss with ARHL identified as the largest modifiable risk factor for dementia^5,9,10^. A large number of studies have found a link between ARHL and the risk of subsequent cognitive decline or dementia and some have suggested that the use of hearing aids can delay or prevent cognitive decline^11–14^. The reasons for the link between the two conditions is currently unknown but various hypothesis have been proposed. These include the following: common pathological mechanisms acting on the auditory pathway and brain such as vascular factors (e.g., diabetes, atherosclerosis, and hypertension)^15,16^; the additional cognitive load required in understanding poor auditory input interferes with other cognitive functions such as language processing and memory^17^; long-term deprivation of auditory inputs may lead to social isolation, depression, and eventually dementia^15^. An alternative explanation might be that hearing loss is an earlier manifestation of reduced cognitive capacity. However, the specific pathogenetic mechanisms underlying these diseases and directionality of the relationship between them are still unknown.

In this work, we aimed to investigate whether there is a shared genetic association between *ARHL* and *AD*, which might explain the link between the two and identify the biology underlying this relationship. Investigation of causal effect of *ARHL* (and other risk factors) on *AD* using statistical genetics methods is an active field of investigation^18–21^. Revealing the reasons which underlie the link might provide an opportunity for therapeutic approaches to both conditions. To this end, we utilized summary statistics from two of the largest genome-wide association studies (GWAS) into adult hearing loss^22^ and late-onset *AD* by the International Genomics of Alzheimer’s Project (IGAP)^23^. We examined the genetic architecture of hearing difficulty and *AD* through recently developed methods in statistical genetics including LD Score regression^24^, genetic correlation^25–27^, partitioned heritability^26,27^ and two-sample Mendelian randomization (MR)^28^. We also screened for mutual genetic risk factors for hearing difficulty and *AD* using risk variants from studies available in MR-base GWAS catalog^28^ using the two-sample MR method. Finally, we conducted multiple gene list functional enrichment analyses to dissect the biological systems underlying genes mapped to the genetic risk variants/single-nucleotide-polymorphisms (SNPs) identified through GWAS for hearing difficulty and *AD*.

## Results

### Summary statistics of *HDiff* with *AD*

We first determined whether the two published GWAS demonstrated overlapping significant loci. Using the two largest GWAS for the respective disorders, late-onset *AD (*Kunkle et. al.)^23^ and *HDiff* (Wells et al.)^22^, there were no shared genome-wide significant SNPs (*P* value <5e-8) or suggestive (*P* value <1e-5) lead SNPs (Supplementary Figure 1). Both the quantile–quantile plots and genomic inflation factors (*λ*_GC_) of the genome-wide test statistic, *λ*_GC_ = 1.31 for *HDiff* cohort, *λ*_GC_ = 1.09 for *AD* cohort demonstrated that *HDiff* GWAS analysis had higher inflation compared with *AD* GWAS even after the GC-correction.

### Genetic correlation between *HDiff* and *AD*

**Fig. 1:**
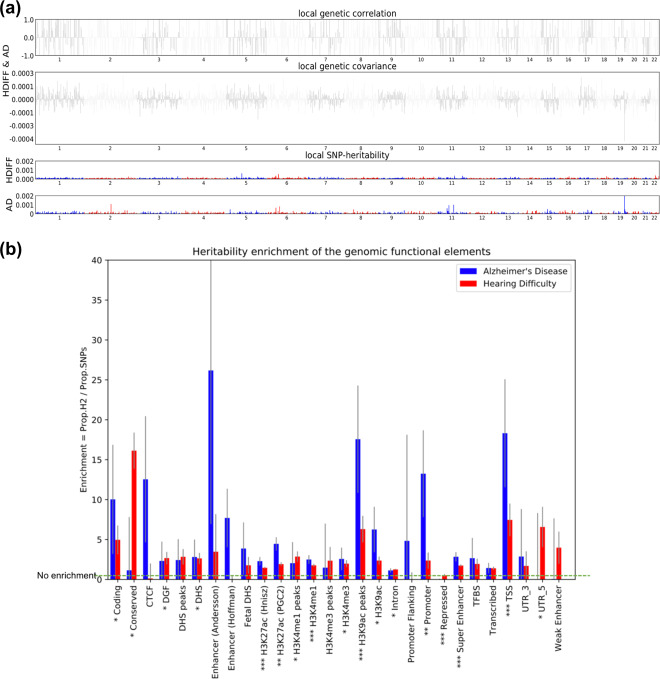
Genomic region-based analysis plots. **a** Manhattan-style plots of local genetic correlation and covariance (top) and local SNP heritability (bottom) for *HDiff* and *AD*. Local genetic correlation corresponds to genomic regions that contribute significantly to the genome-wide genetic correlation. Local genetic covariance corresponds to the similarity between *HDiff* and *AD* driven by genetic variations localized at a specific region in the genome. **b** Heritability enrichment (% of heritability/% of SNP) values in *HDiff* and *AD* SNPs located in each of 27 genomic functional elements. Error bars represent jackknife standard errors around the estimates of enrichment. Key: *CTCF* CCCTC-binding factor, *DGF* digital genomic footprint, *DHS* DNase I hypersensitive site, *TFBS* transcription factor binding site, *TSS* transcription start site, *UTR* untranslated region. Single, double, and triple asterisks indicate significance at *P* < 0.05 after Bonferroni-correction in *HDiff*, *AD*, and both *HDiff* and *AD,* respectively.

Next, we tested whether shared variance between the two disorders has common genetic causes, quantified by the amount of genetic correlation. The total heritability estimated using LD Score (LDSC) for *HDiff* and *AD* is 0.072 (sd = 0.003) and 0.071 (sd = 0.011), respectively. *HDiff* and *AD* exhibited low genetic correlation (*r*_g_ = 0.027, *P* value = 0.659). Removal of chromosome 19, which contains the *APOE* locus, from the analysis did not affect this observation (*r*_g_ = 0.011, *P* value = 0.83). Thus, indicating little or no genetic overlap. Similarly, there was no regional genetic correlation between *HDiff* and *AD* (Fig. 1a) estimated using Heritability Estimation from Summary Statistics (HESS).

### Partitioned heritability

The previous analyses showed a lack of shared genetic loci and lack of genetic correlation between *AD* and *HDiff*. Here, using partitioned heritability, we sought to investigate whether the heritability for *HDiff* and *AD* is enriched in the same genomic functional annotations. The functional annotations include protein-coding genes, their transcription start sites as well as regions of histone modifications that signal the accessibility to the DNA for processing. Partitioned heritability analysis showed significant enrichment (proportion of *h*^*2*^/proportion of SNPs) for both *HDiff* and *AD* in histone modification markers for active enhancers/promoters (e.g., H3K27ac, H3K4me1, H3K9ac peaks), super-enhancer, and transcription start sites (Fig. 1b).

### MR on *HDiff* and *AD*

**Fig. 2:**
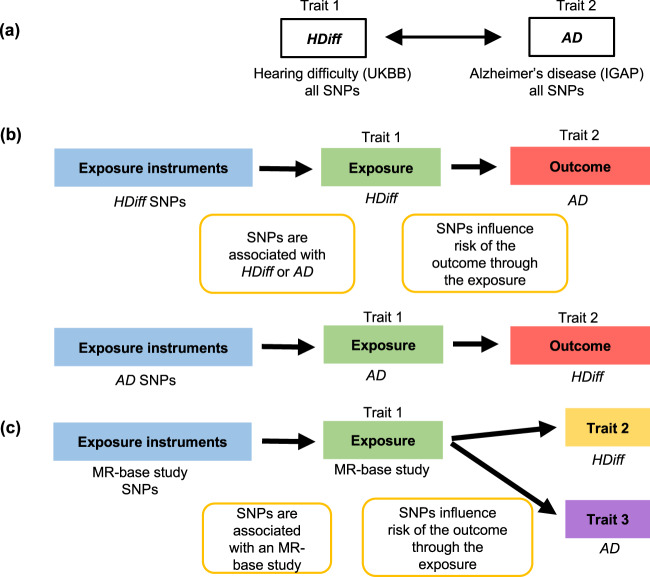
Assumptions of our genetic correlation and Mendelian randomization analyses. **a** Genetic correlation using LD score regression between Hearing difficulty (*HDiff*) and Alzheimer’s disease (*AD*) genome-wide SNPs. **b** Bidirectional Mendelian randomization using exposure instruments (e.g., genome-wide significant lead SNPs from *HDiff* GWAS) in causal association with outcome (e.g., *AD*). **c** Mendelian randomization using SNPs from studies available in MR-base database as exposure instruments in causal association with the outcome for each trait, *HDiff*, and *AD*.

**Fig. 3:**
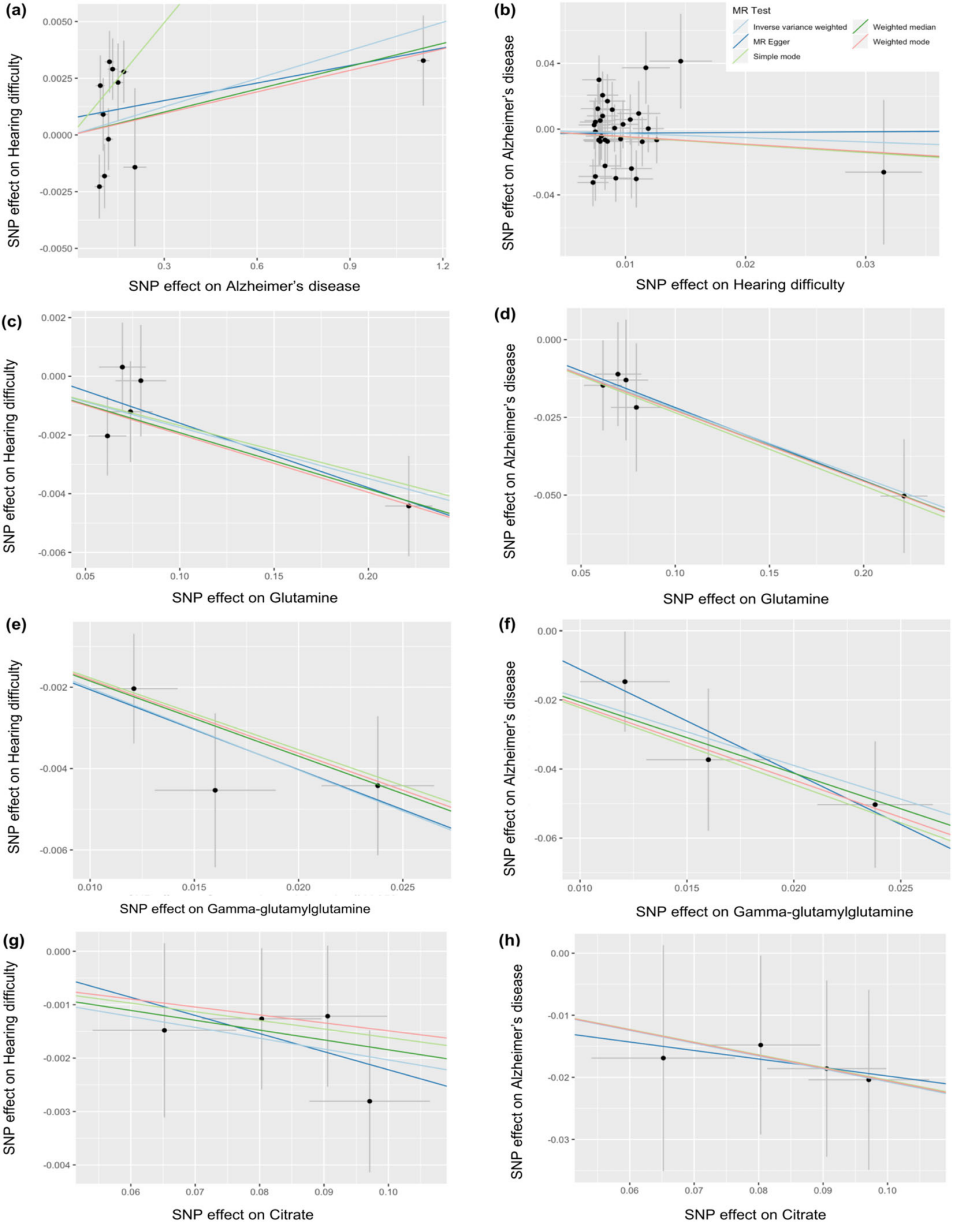
MR regression plots for instrumental SNPs on *x* axis and outcome SNPs on *y* axis. MR regression plots for SNP effect on Hearing difficulty plotted against SNP effect on **a** Alzheimer’s disease, **c** glutamine, **e** gamma-glutamylglutamine, **g** citrate and SNP effect on Alzheimer’s disease plotted against SNP effect on **b** hearing difficulty, **d** glutamine, **f** gamma-glutamylglutamine, **h** citrate. MR regressions were calculated under the Inverse variance weighted, MR-Egger, Weighted median, Simple mode, and Weighted mode approaches.

We used the method of MR to investigate the causal effect of one disease on the other. The causal effects of *HDiff* (termed the “exposure” in an MR analysis) on *AD* (referred to as the “outcome”) and vice versa were investigated using two-sample MR analysis (Fig. 2b). The number of independent genome-wide significant SNPs (termed the “instruments”) were 34 for *HDiff* to *AD* MR analysis and 11 for *AD* to *HDiff* (Supplementary Data 1 and 2) after filtering of SNPs and harmonization step between exposure and outcome SNPs. Using these SNPs, bidirectional MR analyses detected a causal link between *AD* risk variants and increased risk of hearing difficulty (Fig. 3a and Supplementary Table 1). No evidence of a link between *HDiff* risk variants and *AD* was detected (Fig. 3b and Supplementary Table 1). Examination of Fig. 3a suggests this causal link between *AD* genetic risk and *HDiff* is being driven by a single SNP with a large effect on *AD* (rs7256200). That SNP is in strong linkage disequilibrium (LD) with rs429358, which is used to define the E4 allele of the *APOE* gene. Repeating the MR analysis without this SNP abolished the causal link between *AD* risk and *HDiff*, confirming that this link is due to *APOE* variation (data not shown).

We also investigated whether a common “exposure” may affect both *HDiff* and *AD*. Given the rich source of instruments and traits available from studies in MR-Base database^28^, we conducted a post hoc screening for potentially shared causative traits (Fig. 2c). A total of 379 traits met our eligibility criteria (see methods section) and were tested for their influence on *HDiff* and *AD* using five different MR methods (Supplementary Data 3). Among all those traits, we found that levels of metabolites glutamine^29^, gamma-glutamylglutamine^30^, and citrate^30^ were each estimated to be potential causal risk factors for both *HDiff* and *AD* in two or more of the MR methods (Supplementary Table 2 and Fig. 3) although none of the traits survived Bonferroni-correction (adjusting for 379 tested traits). Negative betas for the MR method for these traits to *HDiff* and *AD* (Supplementary Table 2) suggest glutamine, gamma-glutamylglutamine, and citrate have a protective effect on *HDiff* and *AD*, i.e., an increase in levels of those metabolites result in a lower risk of disease.

For all genetic variants associated with glutamine, gamma-glutamylglutamine, and citrate, we extracted corresponding *HDiff* and *AD* summary statistics (Supplementary Table 3), and none of the variants were individually associated with *HDiff* and *AD* risks at the genome-wide significant threshold (*P* value <5e-08). However, the “leave-one-out test” identified that one particular SNP (rs2657879) was driving the causal association for both glutamine and gamma-glutamylglutamine. This SNP is an exonic variant located in *GLS2*, a gene encoding for Glutaminase 2 protein (Supplementary Table 3). Furthermore, SNP rs2040771 was identified as driving the causal association of citrate with *HDiff* and *AD* and the nearest gene to this variant is *SLC25A1* encoding Solute Carrier Family 25 Member 1, a mitochondrial citrate transporter.

### Gene set enrichment analysis

**Fig. 4:**
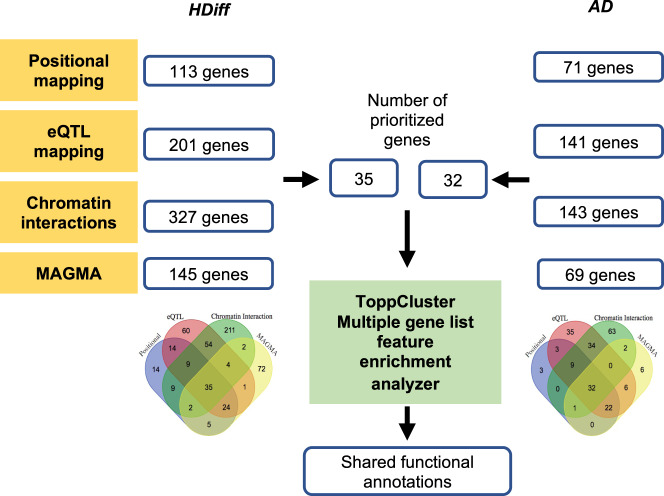
Flow chart for the gene mapping, prioritization, and gene set enrichment analysis. Gene lists of FUMA outputs based on position, eQTL, chromatin interactions, and a gene-based associations (MAGMA) analysis were all compared using Venn diagram for *HDiff* and *AD* each. The Gene set enrichment analysis of the prioritized genes in *HDiff* and *AD* was done using ToppCluster tool producing a list of shared functional enrichment annotations.

**Fig. 5:**
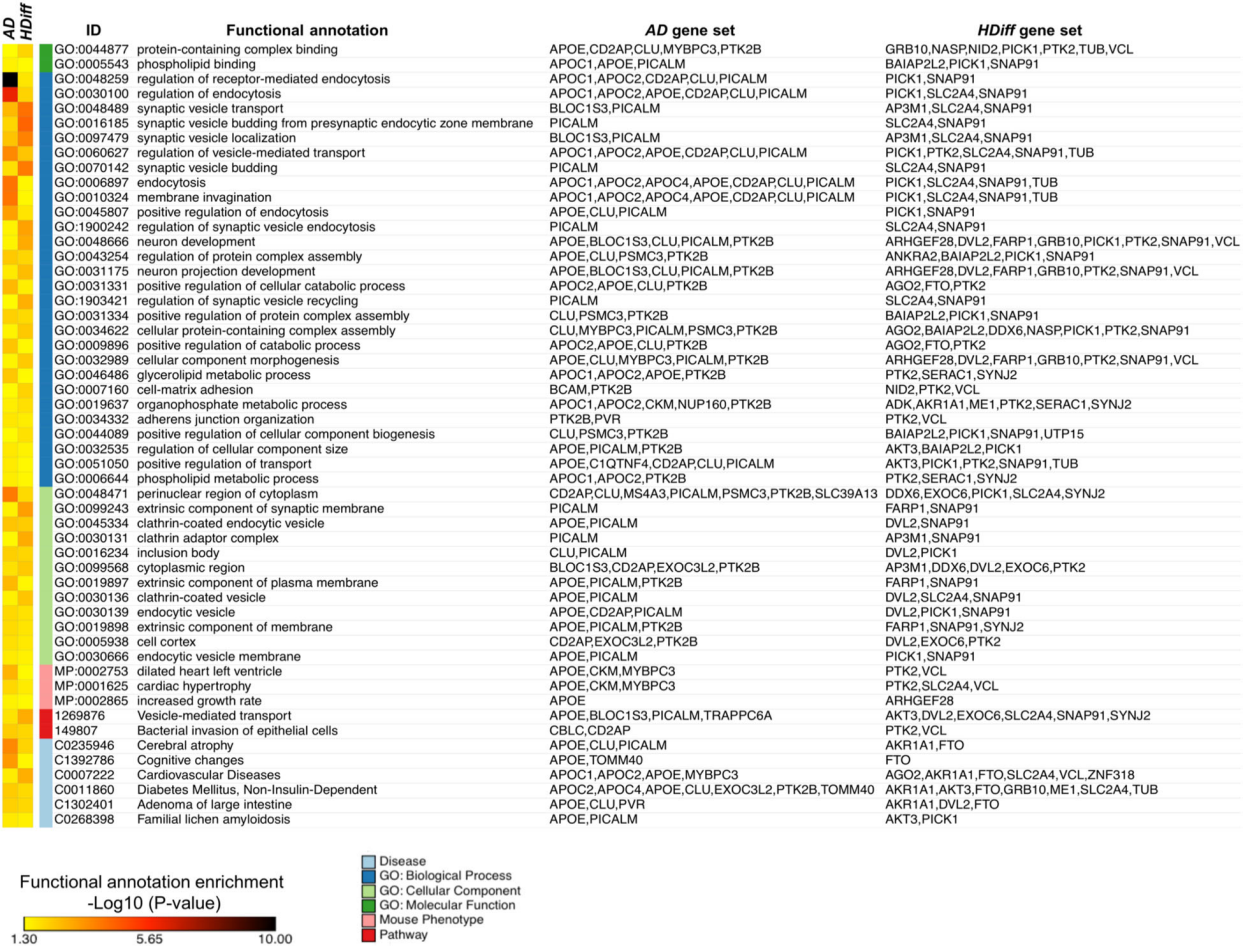
Gene functional enrichment analysis. Functional annotations enriched at uncorrected *P* value <0.05 identified by ToppCluster tool among prioritized *HDiff* and *AD* genes. None of the annotation survived multiple testing corrections at FDR level 0.05, however, interesting functions are enriched even at the nominal level.

Pathway-based analysis in GWAS is widely used to discover gene set functional associations. Gene set enrichment analyses of the resulting *HDiff* and *AD* prioritized gene sets (Fig. 4), showed overlapping functional annotations (Fig. 5). Although owing to the small number of genes, no shared enrichment survived the a false discovery rate (FDR) correction, there were several interesting shared functional annotations at the *P* value <0.05 threshold (Fig. 5). Overall, they shared biological processes such as regulation of endocytosis GO:0030010 and processes related to the neuronal signaling pathway (synaptic vesicle transport GO:0048489, neuron development GO:0048666), cellular component morphogenesis GO:0032989, glycerolipid metabolism GO:0046486, adherens junction organization GO:0034332, cell-matrix adhesion GO:0007160. Genes are also encoded for proteins found at perinuclear region of cytoplasm, extrinsic of synaptic membrane, cell cortex, and clathrin coat vesicles. In addition, we found prioritized genes in *HDiff* and *AD* to also be involved in diseases such as cerebral atrophy, cognitive changes, cardiovascular diseases, diabetes mellitus, familial lichen amyloidosis, and adenoma of large intestine (Fig. 5).

## Discussion

This study investigated whether there is evidence for a shared genetic link between hearing loss and dementia and whether hearing loss is independently or dependently related to the most common form of dementia, AD. Hence, we performed genetic correlation, partitioned heritability, MR, and gene set functional enrichment analysis studies using summary statistics from the largest GWAS available for either disease to date originating from UKBB^22^ and IGAP consortium^23^. The results do not support a broad shared genome-wide genetic architecture between *HDiff* and *AD* that could explain the link between the two conditions. However, results from MR analysis provide some evidence to suggest that AD causes greater hearing difficulty at the level of *AD* genetic risk variants. The strength of these data are nominal as it does not survive multiple testing corrections and is largely being driven by *APOE* but if confirmed this causal link might suggest that hearing loss is an early manifestation of AD rather than hearing loss accelerating dementia or a common pathology being responsible for both diseases. Some studies have previously reported an association between *APOE* isoform and hearing loss in candidate gene studies^31,32^. The recent work by Mitchell et al.^21^ reported a moderate genetic correlation between the genetic *AD*-risk phenotype^33^ and hearing difficulty. Our analysis, which used clinical AD rather than genetic *AD* risk, could not confirm this genetic correlation. Both studies agree that there was no evidence for a direct causative link between AD and ARHL. However, the underestimated effect of APOE-e4 on genetic AD risk (OR = 1.18; Jansen et al.)^33^ compared with clinical AD (OR = 3.32; Kunkle et al.)^23^ may therefore miss the subtle effect of the APOE locus on hearing phenotypes in the MR analysis.

Instead of a shared genetic architecture, we observe non-overlapping gene sets that contributed to the same molecular functions, implying potential shared pathways that contribute to the two disorders. Furthermore, our post hoc systematic screening suggested potential horizontal pleiotropies where one variant has independent effects on multiple traits. This was observed in variants associated with levels of metabolites glutamate, gamma-glutamylglutamine, citrate, and both the *HDiff* and *AD* traits. Both, glutamine and gamma- glutamylglutamine share a causal variant, rs2657879, which is a coding variant located in the *GLS2* gene causing a benign missense mutation according to SIFT and PolyPhen scores provided by the Variant Effect Predictor^34^. The main function of *GLS2* encoded protein Glutaminase 2 is the catalysation of the hydrolysis of glutamine to stoichiometric amounts of glutamate and ammonia^35^.

Glutamate is a key player in maintaining the stability of synaptic signaling and the involved cells including glial cells (e.g., astrocytes) and neurons. Instability in the Glutamatergic pathway usually caused by Glutamate excitotoxicity can hamper the stability of neurons by allowing high levels of Ca2+ influx, thus, activating a number of damaging enzymes and resulting in neuronal cell death^16,36^. Neuronal cell death correlates clinically with the progressive decline in cognition/memory and the development of pathological neural brain atrophy seen in AD patients^37^. It is well-known that in the noise-exposed and aging ear, loss of sensory hair cells results in cochlear nerve fiber degeneration. However, it was recently established that cochlear nerve synapses can be damaged even when hair cells survive, one such pathway is through glutamate excitotoxicity. This underlies hidden hearing loss or synaptopathy^38^ where individuals report difficulties with hearing in background noise despite normal hearing thresholds in pure tone audiograms. Neuronal loss is therefore fundamental in the development of both *HDiff* and *AD*.

On the other hand, little is known about the involvement of citrate in neurodegenerative diseases including ARHL and AD. In addition to the link to Glutamate suggested by the MR analysis, synaptic activity emerged also as one of the shared biological processes from the analysis of prioritized genes in both disorders. Interestingly, the disease enrichment analysis suggested that some of the prioritized genes were the risk genes for developing cerebral atrophy, cognitive changes, cardiovascular diseases, and diabetes mellitus. The latter two are well-recognized risk factors for developing *HDiff* and *AD*^39–41^. High fat and sugar intake can lead to increased free fatty acids and triglycerides and reduced vascular supply to the brain and cochlea, thus promoting hearing loss, brain atrophy, and cognitive declines^42,43^.

Overall, our analysis suggested a possible causal link from *AD* genetic risk to *HDiff*, which was driven by the *APOE* gene. Both disorders appear to share some common underlying molecular processes, however, different genes involved in these processes emerged from recent large GWAS for each of the disorders. As it is not economically viable to perform pure tone audiometry in the number of individuals required to detect genome-wide associations, our analysis utilized self-reported hearing data. This encompasses both peripheral sound detection and central processing of the auditory input so the failure to identify any strong common genetic component is notable. Furthermore, our analyses indicated that the reported correlation between *HDiff* and increased rate of dementia diagnoses may be due to underlying shared upstream risk factors. For instance, the glutamatergic system is central to neurobiology to both *HDiff* and *AD,* and both disorders are linked to genes associated with diabetes and cardiovascular diseases, both of which are risk factors for *HDiff* and *AD*. In conclusion, our genetics-based analysis links the two disorders by shared vulnerability rather than a shared genetic architecture.

## Methods

### Participants

Our analyses were based on the largest publicly available GWAS for adult hearing loss and late-onset AD. The hearing loss GWAS was conducted on subjects from the UK Biobank comprising 250,389 subjects (87,056 cases and 163,333 controls) and 9,740,198 SNPs^22^. Hearing loss in case subjects was defined as subjects responded “Yes” to both questions “Do you have any difficulty with your hearing?” and “Do you find it difficult to follow a conversation if there is background noise?” control subjects were selected if their response to both questions was “No”. Participants included in their study were of above 50 years of age^22^. Here, we used the same hearing loss phenotype representation, *HDiff* throughout this study, and the summary statistics for *HDiff* were obtained from the publicly accessible repository (10.5281/zenodo.3490750). The summary statistics for late-onset *AD* were obtained from The National Institute on Aging Genetics of Alzheimer’s Disease Data Storage Site (NIAGADS), under accession NG00075, the largest *AD* GWAS based on clinical diagnosis (this is opposed to larger studies including parental diagnosis such as Jansen et al.)^33^. Summary statistics were from stage 1 of the GWAS comprising 63,926 subjects (21,982 cases and 41,944 controls) and 11,480,632 SNPs^23^. The analyses presented in this work were solely based on summary statistics obtained from previously published analyses and therefore no ethical approval was required and written informed consents were obtained from all participants in respective studies.

### Heritability and genetic correlation

In brief, heritability represents the degree of variability in a phenotypic trait that comes from genetic differences and genetic correlation refers to the amount of shared heritability between two traits. Estimating SNP-based heritability and genetic correlation from published GWAS summary statistics is important to investigate the genetic architecture of disorders and to explore relationships between disorders. An LD score regression was employed to quantify the shared genetic architecture between *HDiff* and *AD* (Fig. 2a). We used the implementation in the LDSC software^24^ and followed a standard pre-processing pipeline: summary statistics for *HDiff* and *AD* were restricted to well-imputed HapMap3 SNPs; pre-computed LD Scores using 1000 Genomes European data from 1000 Genomes Project were downloaded; input files in the corrected format were used to run LD Score regression; heritability and genetic correlation were estimated. LD Score regression computes the genetic correlation across the entire genome. In a complementary analysis, we computed also the regional genetic correlation^27^ and regional heritability^44^ using the HESS method.

### Partitioned heritability

Some functional categories of the genome contribute disproportionately to the total heritability of a complex trait. Partitioned heritability enables us to estimate the amount of heritability that is associated with specific genomic annotations. For example, this allows us to answer what amount of the total heritability is located in protein-coding genes. We used partitioned heritability to investigate whether the heritability for *HDiff* and *AD* are enriched in the same genomic functional annotations. Total heritability, *h*^2^, partitioned by functional annotations were calculated using LDSC^26^. The European population of the 1000 Genomes project was used as a reference panel.

### Mendelian Randomisation

MR is a method that uses genetic variant associations for detecting evidence of causal relationship between two traits. We performed MR to investigate the causal relationship between *HDiff* and *AD*. In order to establish causality, MR uses SNPs strongly associate with exposure (e.g., *HDiff*) as instrumental variables and tests whether these SNPs are jointly affecting the other disease or trait (e.g., *AD*). Instrumental variables are only valid if these key assumptions are fulfilled: (i) they associate with the exposure of interest, (ii) share no common cause with the outcome and (iii) do not affect the outcome except through the exposure^45^. MR focuses on few selected SNPs, typically exceeding genome-wide significance levels, and is therefore conceptually different from genetic correlation, which considers the entire genome. Bidirectional causal effects were estimated between *HDiff* and *AD* using two-sample MR implemented in the MR-base platform (TwoSampleMR v0.4.26)^28^ in R, see Fig. 2b.

We used SNPs that were independently associated with the potentially causal trait, either *HDiff* (41 SNPs) or *AD* (14 SNPs) at genome-wide significant level (*P* value <5e-8) that serve as instruments in the MR analysis. Instrument SNPs were filtered to SNPs available in the outcome, followed by harmonization of the instrument SNPs and SNPs in outcome resulted in the removal of ambiguous, palindromic, and not inferable SNPs. For the MR analysis from *HDiff* to *AD*, 7 SNPs were removed after filtering and harmonization steps, resulted in 34 *HDiff* SNPs and 3 SNPs were removed after the harmonization step for reversed MR analysis, resulted in 11 *AD* SNPs. We then performed MR analysis on the resulting exposure and outcome SNPs (Supplementary Data 1 and 2).

The analysis was done using various MR methods available in MR-base (i.e., inverse variance weighted (IVW), MR-Egger, weighted median, simple mode, and weighted mode) where each method provided an estimate of the causal effect of exposure on the outcome. IVW is the conventional MR method that utilized combined ratio estimates from each SNP into an overall estimate. In weighted median, weights are given to the ordered ratio estimates, and the sum of weights is 1. In MR-Egger, the set of instrument-outcome effect sizes is regressed upon the set of instrument-exposure effect sizes and weighting the regression as in IVW. However, MR-Egger does not constrain the intercept at the origin thus, making it a more flexible method that could deal with invalid instruments^46,47^. Meanwhile, simple and weighted mode using the mode of the unweighted and IVW empirical density function, respectively, as the causal effect estimate^48^.

In order to screen for potentially shared causes between *HDiff* and *AD*, in a post hoc analysis, we conducted a systematic MR analysis (Fig. 2c), where all of the available instruments from MR-base were utilized. The *HDiff* GWAS used samples from UK Biobank, thus to avoid biased estimates due to (partial) sample overlap, traits were removed if their GWAS included samples from UK Biobank. In addition, traits with instruments comprising only one SNP were removed. MR regressions for 379 traits with instruments mappable to *HDiff* and *AD* were analyzed (Supplementary Data 3). Causal effects that are significant at a nominal *P* value of 0.05 were reported. Leave-one-out analysis was conducted to determine whether a variant strongly contributes to the observed significant relationship.

### Gene mapping, prioritization, and functional enrichment analysis

We sought to investigate whether genes implicated by the recent GWAS on *HDiff*^22^ and *AD*^23^ are either shared or share a common biological pathway. To this end, we first needed to convert locations with significant SNPs to corresponding genes. We followed three complementary strategies (Fig. 4): first, positional mapping was used to map SNPs to genes based on physical distance, if they were within 10 kb from a known protein gene. Second, expression quantitative trait loci (eQTL) mapping was used by mapping SNPs to genes if the allele variation at the SNP is associated with expression levels of a gene utilizing information of different tissue types from databases (GTEx v8, Blood eQTL, BIOS QTL, PsychENCODE, MuTHER, BRAINEAC). Genes with significant eQTL associations at a FDR of 0.05 were considered. Finally, Chromatin interaction mapping was carried out by mapping SNPs to genes when there is a three-dimensional DNA-DNA interaction between the SNP and gene. We used the web-based platform FUMA (Functional Mapping and Annotation of Genome-Wide Association Studies)^49^ all three strategies. FUMA parameters for chromatin interactions include, all HiC built-in chromatin interaction data, FDR threshold of 1e-6, 250 bp upstream and 500 bp downstream from TSS as promoter region window, interaction overlapped with a predicted enhancer region in any of the 111 tissue/cell types found in the Roadmap Epigenomics Project.

Genes mapped using these three strategies were combined with gene-based statistics computed by MAGMA^50^ from summary statistics. This method was accessed through the FUMA web service. MAGMA combines SNP association P-values within or in close proximity to protein-coding genes to derive a *P* value describing the association of that gene. A genome-wide significance was defined at *P* = 0.05/19,201 = 2.6e-6). Each of the four strategies produced a list of genes. A Venn diagram was used to retrieve overlapping genes between four gene lists (Fig. 4). A comparison of functional enrichments for *HDiff* and *AD* prioritized gene lists was carried out using the ToppCluster suite^50,51^. Gene Ontology (GO) terms for molecular function, biological process, cellular component, pathways, human and mouse phenotypes, and disease annotation options were selected for enrichment analysis. ToppCluster reported the enrichment P-values at both uncorrected and corrected levels. Enrichment of features at uncorrected level is useful for a smaller number of genes input and for exploratory analysis. GO terms with uncorrected *P* value <0.05 in both disorders were considered for discussion.

### Reporting summary

Further information on research design is available in the Nature Research Reporting Summary linked to this article.

## Supplementary information

Supplementary Information

Supplementary Data 1-3

Reporting summary

## Data Availability

The summary statistics for *HDiff* are available from the publicly accessible repository (10.5281/zenodo.3490750). The summary statistics for late-onset *AD* were obtained from The National Institute on Aging Genetics of Alzheimer’s Disease Data Storage Site (NIAGADS), under accession NG00075. All other data supporting the findings of this study are available within the paper and its supplementary information files.

## Code availability

The HESS method was used to compute regional genetic correlation (https://huwenboshi.github.io/hess/local_rhog/) and regional heritability ((https://huwenboshi.github.io/hess/local_hsqg/). Partitioned heritability by functional category was done according to (https://github.com/bulik/ldsc/wiki/Partitioned-Heritability) using LDSC v1.0.1 (https://github.com/bulik/ldsc). Genetic correlations were calculated using LD score (LDSC v1.0.1, https://github.com/bulik/ldsc). MR analyses between *HDiff*, *AD*, and all other traits in MR-base platform (https://mrcieu.github.io/TwoSampleMR/) were done using two-sample MR (TwoSampleMR0.4.26) in R version 4.0.0. Manhattan plot and identification of lead SNPs were done using Functional Mapping and annotation of genome-wide association studies (https://fuma.ctglab.nl/). A comparison of functional enrichments for *HDiff* and *AD* prioritized gene lists was carried out using the ToppCluster suite (https://toppcluster.cchmc.org/).
