# Supplementary material for: Hearing difficulty is linked to Alzheimer’s disease by common genetic vulnerability, not shared genetic architecture
Source: NPJ Aging Mech Dis. 2021 Jul 22;7:17. doi: 10.1038/s41514-021-00069-4 (PMC8298411; doi:10.1038/s41514-021-00069-4)
Supplement: Supplementary file 1 — Supplementary Information [file 41514_2021_69_MOESM1_ESM.pdf]

Hearing difficulty is linked to Alzheimer's disease by common genetic vulnerability, not shared genetic architecture

Fatin N. Zainul Abidin[a,b], Helena R. R. Wells[a,c], Andre Altmann[b*], Sally J. Dawson[a*]

a UCL Ear Institute, University College London, London, UK.
b Centre for Medical Image Computing, Department of Medical Physics and Biomedical Engineering, University College London, London, UK.
c Department of Twin Research and Genetic Epidemiology, King's College London, London, UK.
* Joint senior authors

Corresponding author at:
UCL Ear Institute, University College London, 332 Gray's Inn Road, WC1X 8EE, London, UK.
Tel: +44 (0)20 7679 8935
E-mail address: sally.dawson@ucl.ac.uk (Sally Dawson)

Supplementary Tables and Data

Supplementary Figure 1	Manhattan plots displaying GWAS results for HDiff and AD	2
Supplementary Table 1	Two-sample bidirectional Mendelian randomization of HDiff and AD	3
Supplementary Table 2	Shared causal traits for HDiff and AD	4
Supplementary Table 3	Characteristics of genetic variants as instruments from glutamine, gamma-glutamylglutamine, and citrate	5
Supplementary Data 1	34 instruments (HDiff SNPs) for Mendelian randomisation analysis of HDiff as exposure	.xls
Supplementary Data 2	11 instruments (AD SNPs) for Mendelian randomisation analysis of AD as exposure and HDiff as outcome	.xls
Supplementary Data 3	379 MR-base instruments association with HDiff and AD	.xls

(a)

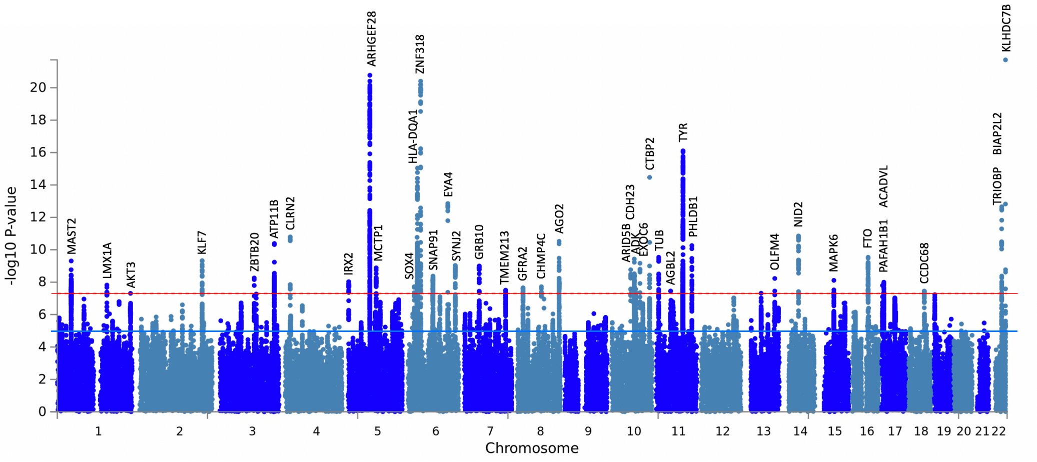

(b)

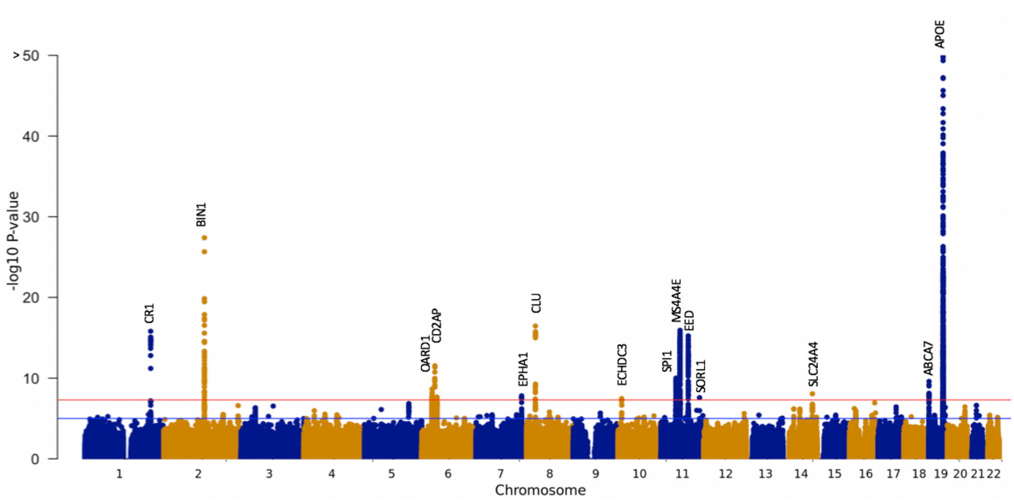

Supplementary Figure 1| Manhattan plots displaying GWAS results for (a) UKBB Hearing difficulty (*HDiff*), and (b) Stage 1 IGAP late-onset Alzheimer's disease (*AD*) phenotypes. The Manhattan plots display the P-values of all SNPs tested in discovery analysis from (Kunkle et al., 2019; Wells et al., 2019). The threshold for genome-wide significance (P-value <5e-08) is indicated by a red solid horizontal line and the genome-wide suggestive threshold (P-value <1e-05) is indicated by a blue horizontal solid line. Loci that reached genome-wide significance are annotated with gene symbol. The inflation factors, λ for *HDiff* and *AD* are 1.31 and 1.09 respectively.

Supplementary Table 1|

Two-sample bidirectional Mendelian randomization of Hearing difficulty and Alzheimer's disease.

Exposure	Outcome	MR method	nSNP	Beta	SE	P-value
HDiff	*AD*	IVW	34	-0.261	0.304	0.390
		MR Egger	34	0.042	1.362	0.975
		Simple Mode	34	-0.479	0.816	0.561
		Weighted Median	34	-0.464	0.424	0.274
		Weighted mode	34	-0.455	0.707	0.524
AD	*HDiff*	IVW	11	0.004	0.002	0.061
		MR Egger	11	0.003	0.003	0.382
		Simple Mode	11	0.016	0.008	0.071
		Weighted median	11	0.003	0.002	0.044
		Weighted mode	11	0.003	0.002	0.080

Key: *AD*, Alzheimer's disease; H*Diff*, Hearing difficulty; IVW, Inverse variance weighted; MR, Mendelian randomization; SE, Standard error.

Supplementary Table 2|

Shared causal traits for Hearing difficulty and Alzheimer's disease. Two-sample Mendelian randomization of glutamine, gamma-glutamyl glutamine, and citrate as exposure and Hearing difficulty and Alzheimer's disease as outcome.

		HDiff				*AD*			
**Exposure**	**MR method**	**nSNPs**	**Beta**	**SE**	**Pval**	**nSNPs**	**Beta**	**SE**	**Pval**
GGG	IVW**	3	-0.202	0.054	0.000	3	-1.948	0.578	0.001
GGG	MR Egger	3	-0.197	0.186	0.482	3	-2.996	1.993	0.374
GGG	Simple mode	3	-0.177	0.077	0.148	3	-2.224	0.746	0.096
GGG	Weighted median**	3	-0.185	0.063	0.003	3	-2.060	0.673	0.002
GGG	Weighted mode	3	-0.181	0.072	0.129	3	-2.160	0.782	0.110
Gln	IVW**	5	-0.017	0.006	0.006	5	-0.222	0.069	0.001
Gln	MR Egger	5	-0.022	0.012	0.172	5	-0.235	0.132	0.175
Gln	Simple mode	5	-0.017	0.012	0.237	5	-0.235	0.120	0.122
Gln	Weighted median**	5	-0.019	0.007	0.007	5	-0.227	0.072	0.002
Gln	Weighted mode	5	-0.020	0.008	0.058	5	-0.228	0.084	0.052
Citrate	IVW**	4	-0.020	0.008	0.012	4	-0.207	0.088	0.018
Citrate	MR Egger	4	-0.034	0.062	0.639	4	-0.137	0.684	0.860
Citrate	Simple mode	4	-0.016	0.014	0.319	4	-0.204	0.111	0.165
Citrate	Weighted median**	4	-0.018	0.009	0.047	4	-0.207	0.103	0.045
Citrate	Weighted mode	4	-0.015	0.011	0.275	4	-0.206	0.114	0.170

Key: *AD*, Alzheimer's disease; *HDiff*, Hearing difficulty; IVW, Inverse variance weighted; Gln, glutamine; GGG, gamma-glutamyl glutamine; SE, Standard error. Beta < 0 means negative effect/protective on the outcome (decreased glutamine, gamma-glutamylglutamine, and citrate) and Beta > 0 means positive effect on the outcome (increased glutamine, gamma-glutamylglutamine and citrate). ** MR methods that are significant for both *HDiff* and *AD* at uncorrected P-value of 0.05

Supplementary Table 3|

Characteristics of genetic variants as instruments from glutamine, gamma-glutamylglutamine, and citrate GWAS with corresponding Hearing difficulty and Alzheimer's disease GWAS data sets. The effect of assessed allele at each locus is indicated by beta.

SNP	Chr	Nearby gene	EA	NEA	EAF	Glutamine GWAS			*HDiff* GWAS			*AD* GWAS		
N=24,462						Beta	SE	P-value	Beta	SE	P-value	Beta	SE	P-value
rs1260326	2	GCKR*	C	T	0.638	0.062	0.010	9.87E-10	0.002	0.001	0.13	8.0E-04	0.016	0.96
rs6729711	2	GLS	A	G	0.181	-0.069	0.012	2.23E-08	0.000	0.002	0.84	0.011	0.018	0.54
rs12306007	12	SLC38A4	T	C	0.140	0.079	0.013	3.63E-09	0.000	0.002	0.94	-0.017	0.025	0.49
**rs2657879**	**12**	**GLS2***	**G**	**A**	**0.176**	**-0.221**	**0.012**	**3.30E-70**	**-0.004**	**0.002**	**9.6E-03**	**0.047**	**0.021**	**0.024**
rs7078003	10	HOGA1	T	C	0.195	0.074	0.011	2.96E-10	0.001	0.002	0.48	-0.022	0.023	0.035
						GGG GWAS			*HDiff* GWAS			*AD* GWAS		
N=7,662						Beta	SE	P-value	Beta	SE	P-value	Beta	SE	P-value
rs1260326	2	GCKR*	T	C	0.412	-0.012	0.002	5.61E-09	0.002	0.001	0.13	0.001	0.016	0.96
rs1751956	10	LYZL1	T	G	0.864	0.016	0.002	2.35E-08	0.000	0.002	0.94	-0.017	0.025	0.49
**rs2657879**	**12**	**GLS2***	**A**	**G**	**0.826**	**0.024**	**0.002**	**5.85E-09**	**-0.004**	**0.002**	**9.6E-03**	**0.047**	**0.021**	**0.024**
						Citrate GWAS			*HDiff* GWAS			*AD* GWAS		
N=24,770						Beta	SE	P-value	Beta	SE	P-value	Beta	SE	P-value
rs1468269	22	GSC2	A	G	0.2613	-0.065	0.011	5.04E-09	0.001	0.001	0.71	0.017	0.018	0.353
rs172642	17	SLC13A5	C	A	0.481	-0.080	0.009	4.80E-18	0.001	0.001	0.34	0.015	0.014	0.304
rs2040771	22	SLC25A1	T	C	0.476	-0.091	0.009	1.28E-22	-0.001	0.001	0.36	0.019	0.014	0.192
rs2921604	5	ANKH	C	T	0.444	0.097	0.094	3.65E-25	0.003	0.001	0.035	0.020	0.014	0.161

Key: *AD*, Alzheimer's disease; *HDiff*, Hearing difficulty; EA, effect allele; NEA, non-effect allele; GGG, gamma-glutamylglutamine; SE, Standard error; SNPs in bold are single SNP that driving the association and influencing the causal relationship using leave one out test * Exonic SN
