# Supplementary material for: Hearing difficulty is linked to Alzheimer’s disease by common genetic vulnerability, not shared genetic architecture
Source: NPJ Aging Mech Dis. 2021 Jul 22;7:17. doi: 10.1038/s41514-021-00069-4 (PMC8298411; doi:10.1038/s41514-021-00069-4)
Supplement: Supplementary file 3 — Reporting summary [file 41514_2021_69_MOESM3_ESM.pdf]

nature research

Corresponding author(s): Sally Dawson

Last updated by author(s): Mar 25, 2021

Reporting Summary

Nature Research wishes to improve the reproducibility of the work that we publish. This form provides structure for consistency and transparency in reporting. For further information on Nature Research policies, see our Editorial Policies and the Editorial Policy Checklist.

Statistics

For all statistical analyses, confirm that the following items are present in the figure legend, table legend, main text, or Methods section.

n/a	Confirmed	
☐	☒	The exact sample size (*n*) for each experimental group/condition, given as a discrete number and unit of measurement
☐	☒	A statement on whether measurements were taken from distinct samples or whether the same sample was measured repeatedly
☐	☒	The statistical test(s) used AND whether they are one- or two-sided<br>*Only common tests should be described solely by name; describe more complex techniques in the Methods section.*
☐	☒	A description of all covariates tested
☐	☒	A description of any assumptions or corrections, such as tests of normality and adjustment for multiple comparisons
☐	☒	A full description of the statistical parameters including central tendency (e.g. means) or other basic estimates (e.g. regression coefficient) AND variation (e.g. standard deviation) or associated estimates of uncertainty (e.g. confidence intervals)
☐	☒	For null hypothesis testing, the test statistic (e.g. *F*, *t*, *r*) with confidence intervals, effect sizes, degrees of freedom and *P* value noted<br>*Give P values as exact values whenever suitable.*
☒	☐	For Bayesian analysis, information on the choice of priors and Markov chain Monte Carlo settings
☒	☐	For hierarchical and complex designs, identification of the appropriate level for tests and full reporting of outcomes
☐	☒	Estimates of effect sizes (e.g. Cohen's *d*, Pearson's *r*), indicating how they were calculated

Our web collection on statistics for biologists contains articles on many of the points above.

Software and code

Policy information about availability of computer code

Data collection	Published summary statistics were used from UK Biobank hearing difficulty GWAS and Alzheimer's disease GWAS papers
Data analysis	The Heritability Estimation from Summary Statistics (HESS) method was used to compute regional genetic correlation (https://huwenboshi.github.io/hess/local_rhog/) and regional heritability ((https://huwenboshi.github.io/hess/local_hsqg/). Partitioned heritability by functional category was done according to (https://github.com/bulik/ldsc/wiki/Partitioned-Heritability) using LDSC v1.0.1 (https://github.com/bulik/ldsc). Genetic correlations were calculated using LD score (LDSC v1.0.1, https://github.com/bulik/ldsc). Mendelian randomization analyses between HDiff, AD and all other traits in MR-base platform (https://mrcieu.github.io/TwoSampleMR/) were done using two-sample Mendelian randomization (TwoSampleMR0.4.26) in R version 4.0.0. Manhattan plot and identification of lead SNPs were done using Functional Mapping and annotation of genome-wide association studies (FUMA, https://fuma.ctglab.nl/). A comparison of functional enrichments for HDiff and AD prioritized gene lists was carried out using the ToppCluster suite (https://toppcluster.cchmc.org/).

For manuscripts utilizing custom algorithms or software that are central to the research but not yet described in published literature, software must be made available to editors and reviewers. We strongly encourage code deposition in a community repository (e.g. GitHub). See the Nature Research guidelines for submitting code & software for further information.

Data

Policy information about availability of data

All manuscripts must include a data availability statement. This statement should provide the following information, where applicable:

- Accession codes, unique identifiers, or web links for publicly available datasets
- A list of figures that have associated raw data
- A description of any restrictions on data availability

HDiff and AD GWAS summary statistics are available from the original GWAS papers and all other data supporting the findings of this study are available within the paper and its supplementary information files.

Field-specific reporting

Please select the one below that is the best fit for your research. If you are not sure, read the appropriate sections before making your selection.

☒ Life sciences ☐ Behavioural & social sciences ☐ Ecological, evolutionary & environmental sciences

For a reference copy of the document with all sections, see nature.com/documents/nr-reporting-summary-flat.pdf

Life sciences study design

All studies must disclose on these points even when the disclosure is negative.

Sample size	Samples were chosen on the basis of the largest available well phenotype GWAS into the two phenotypes, hence with the greatest power.
Data exclusions	None
Replication	Replication was not attempted as part of this discovery study
Randomization	Samples were allocated based on phenotypes
Blinding	Blinding was not done because subjective bias is not an issue in this type of analysis, which is essentially objective.

Reporting for specific materials, systems and methods

We require information from authors about some types of materials, experimental systems and methods used in many studies. Here, indicate whether each material, system or method listed is relevant to your study. If you are not sure if a list item applies to your research, read the appropriate section before selecting a response.

Materials & experimental systems

n/a	Involved in the study	
☒	☐	Antibodies
☒	☐	Eukaryotic cell lines
☒	☐	Palaeontology and archaeology
☒	☐	Animals and other organisms
☐	☒	Human research participants
☒	☐	Clinical data
☒	☐	Dual use research of concern

Methods

n/a	Involved in the study	
☒	☐	ChIP-seq
☒	☐	Flow cytometry
☒	☐	MRI-based neuroimaging

Human research participants

Policy information about studies involving human research participants

Population characteristics	This is a secondary analysis of published date which can be found in the original citations
Recruitment	This is a secondary analysis of published data which can be found in the original citations
Ethics oversight	As this was a secondary analysis of summary statistics from previous studies no ethical permission was required

Note that full information on the approval of the study protocol must also be provided in the manuscript.
